# A Scalable Solution Route to Porous Networks of Nanostructured Black Tungsten

**DOI:** 10.3390/nano11092304

**Published:** 2021-09-05

**Authors:** V. Vinay K. Doddapaneni, Kijoon Lee, Tyler T. Colbert, Saereh Mirzababaei, Brian K. Paul, Somayeh Pasebani, Chih-Hung Chang

**Affiliations:** 1 School of Chemical, Biological and Environmental Engineering, Oregon State University, Corvallis, OR 97331, USA; doddapav@oregonstate.edu (V.V.K.D.); tycolbert94@gmail.com (T.T.C.); 2 School of Mechanical, Industrial and Manufacturing Engineering, Oregon State University, Corvallis, OR 97331, USA; leekij@oregonstate.edu (K.L.); mirzabas@oregonstate.edu (S.M.); brian.paul@oregonstate.edu (B.K.P.); somayeh.pasebani@oregonstate.edu (S.P.); 3 Advanced Technology and Manufacturing Institute (ATAMI), Corvallis, OR 97330, USA

**Keywords:** nanostructures, black tungsten, solution-based, solar absorber

## Abstract

This paper studied the feasibility of a new solution-processed method to manufacture black tungsten nanostructures by laser conversion of tungsten hexacarbonyl precursor on the Inconel 625 substrate under argon atmosphere at ambient pressure. The results show that sublimation of the precursor can be prevented if the decomposition temperature (>170 °C) is achieved using the laser heating method. Three different laser powers from 60–400 W were used to investigate the role of laser parameters on the conversion. It was found that lower laser power of 60 W resulted in a mixture of unconverted precursor and converted tungsten. Higher laser powers >200 W resulted in α-W (BCC) in one step without further heat treatment. Different oxygen concentrations from 0.5 ppm to 21 vol% were used in the laser canister to investigate the effect of oxygen concentration on the conversion. It was found that the hard vacuum (>10^−4^ torr) or hydrogen is not necessary to obtain α-W (BCC). The solar absorptance varied from 63–97%, depending on the amount of precursor deposited on the substrate and oxygen content in the laser canister. This solution-based laser conversion of tungsten precursor is a scalable method to manufacture tungsten coatings for high-temperature applications.

## 1. Introduction

Tungsten, the highest melting point element known in the periodic table, with low thermal expansion coefficient, good thermal and electrical conductivity, wear-resistance, and chemical stability, has a wide range of applications, such as ohmic contacts, interconnects, solar thermal absorbers, IR reflectors, diffusion barriers, and high-strength metal matrix composites [1,2,3,4]. One growing use of W is as a high-temperature, absorber layer to increase the solar absorptivity and decrease the thermal emissivity of solar thermal collectors or receivers for various energy applications [1,5,6,7]. Particularly, creating plasmonic nanostructured metals (nanorods, nanoparticles, nanoporous films) is of great interest as they augment the absorption of electromagnetic radiation over a tunable range of UV to near IR [8,9]. Different synthesis methods have been used to manufacture W films and coatings capable of modifying the solar absorptance of these surfaces. Gesheva et al. [10] deposited black W films by chemical vapor deposition (CVD) of tungsten hexacarbonyl (W(CO)_6_) under hydrogen atmosphere for photothermal solar energy conversion applications. Shah et al. [9] manufactured a spectrally selective solar absorber by laser sintering of W micro and nanoparticles on stainless steel (SS) substrate with a solar absorptance of 83% and emissivity of 11.6%. Sibin et al. [4] manufactured IR-reflective W films by magnetron sputtering to control the thermal emittance of stainless steel substrates. Gao et al. [1] produced surface textured W with enhanced absorption of 74% and reduced emittance of 5%. They observed increased solar absorptance of 0.9 with SS/W/Al_2_O_3_ tandem coatings. Salama et al. [11] developed a better gamma shielding capability of SS316 alloy by replacing Mo with W.

Paul et al. [12] developed a hybrid metal additive manufacturing process that exploits liquid ink-jetting (drop on demand) into the powder bed to manufacture metal alloys. This method is capable of direct printing of alloys that are difficult to process via conventional powder metallurgy routes such as oxide dispersion strengthened alloys. It can also manufacture materials with spatially tailored properties (such as functionally graded alloys). Producing W-based metal matrix composites such as W-austenitic SS [11], W-reinforced Al matrix [2], and Cu-W composites [13,14] by hybrid metal additive manufacturing (AM) would be very beneficial for a wide range of applications.

Despite the potential for utilizing W films, coatings, and patterns for a wide variety of applications, there are very few studies of solution processing methods of W films and coatings. One such study produced metallic W films by spin coating peroxopolytungstic acid [15]. This spin coating method indirectly produces W by reducing the tungsten oxide films made from the precursor. Nonetheless, the implementation of scalable manufacturing is a significant challenge. Aerosol jet printing (AJP) is an AM technique that enables the large-scale manufacturing of films, coatings, and devices. It is a versatile method that uses inks with a wide range of rheological properties [16].

Nevertheless, a significant challenge to develop inks containing W is the precursor solubility and their reaction chemistry. Some of the organometallic precursors being used for CVD and atomic layer deposition (ALD) approaches to depositing W films include tungsten hexafluoride (WF_6_) and tungsten hexachloride (WCl_6_). However, they require strong reducing agents such as H_2_ and silane gas to reduce these halide precursors to metallic tungsten. In addition, the byproduct gases are corrosive and thus not desirable. W(CO)_6_ is another widely used metal-organic precursor because of its volatile nature, and it decomposes into W and CO gas at temperatures >170 °C in an inert atmosphere. It has been a precursor choice to produce W films by CVD and laser-induced CVD [3,10,17].

This paper presents the feasibility of using a molecular precursor (W(CO)_6_) ink dissolved in a solvent to manufacture nanostructured W coatings using laser conversion. This new solution-based method entails scalability, pre-printing the precursor patterns onto a surface of interest, and converting it later using the laser. Additionally, this process opens avenues to manufacturing W-based functionally graded materials and tailored alloys using hybrid metal additive manufacturing processes. Prior to the printing, the feasibility of converting the sublimable precursor directly without sublimating the precursor was studied. Morphology, crystal structure, composition, and solar absorptance of produced W coatings were also analyzed.

## 2. Materials and Methods

### 2.1. Materials

Tungsten hexacarbonyl (99% pure, 0.3% Mo, Strem Chemicals) and tetrahydrofuran (THF, >99.9%, fisher chemical) were used as a precursor and solvent, respectively. We prepared 0.071 M ink by adding the precursor to THF and ultrasonicated for 15 min. Inconel 625 substrates, purchased from McMaster-Carr, were polished using emery paper and cleaned with acetone, methanol, and DI water before depositing the precursor.

### 2.2. Deposition of Precursor and Laser-Assisted Conversion

The deposition was carried through a Sonozap ultrasonic atomizer connected with a new era syringe pump system and griffin motion stage to control. The syringe pump was programmed to deliver the same volume each time with a flow rate of 5 mL/h, and the movement of the substrate was controlled in one direction using the stage during the printing and in other directions manually. The substrate was heated to 40 °C using an external heater to remove solvent during the printing.

**Figure 1:**
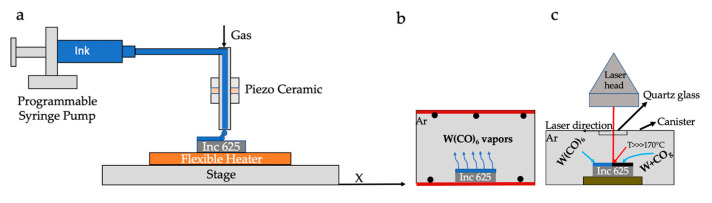
Schematics of experimental setup (**a**) precursor deposition setup; (**b**) precursor deposited substrate placed in a conventional furnace; (**c**) laser canister setup used in this work.

The printed precursor was then converted using a 1000 W Rofin FL010 laser welder (1080 nm). An inert atmosphere was provided during the laser conversion by placing the precursor inside a vacuum canister that consists of a vacuum flange sealed with an IR-transparent quartz plate and permits the ultra-high purity argon (99.999%). Different laser beam powers (P) of 60, 200, and 400 W at a scan speed of 1000 mm/s were used to study the conversion of the precursor. Laser power of 200 W was used to investigate the effect of atmosphere and precursor amount because no significant change in the reflectance of samples manufactured at 200 W and 400 W was observed. Figure 1a,c show the schematics of the deposition setup and the laser conversion setup, respectively. Table 1 shows the experimental conditions. 

### 2.3. Characterization Methods

Thermogravimetry analysis (TGA, TA instruments Q500) was used to study the effect of heating rate on the precursor. An X-ray diffractometer (XRD, Bruker-D8, Cu-Kα radiation) was used to examine the crystal structure of as-converted and annealed coatings. Scanning electron microscopy (S.E.M., Quanta 600) and transmission electron microscopy (TEM/STEM, FEI Titan 80-200) were used to analyze the nanostructured W coatings morphologies, size, and composition. The focused ion beam lift-out technique (FIB, FEI Helios 650 dual beam) was used to make samples for TEM/STEM analysis. UV-VIS-NIR spectroscopy (JASCO V-670 with an integrated sphere) was used to measure the reflectance of the nanostructured W over the wavelength range of 280 to 2500 nm.

## 3. Results and Discussion

### 3.1. Thermogravimetric Analysis (TGA)

**Figure 2:**
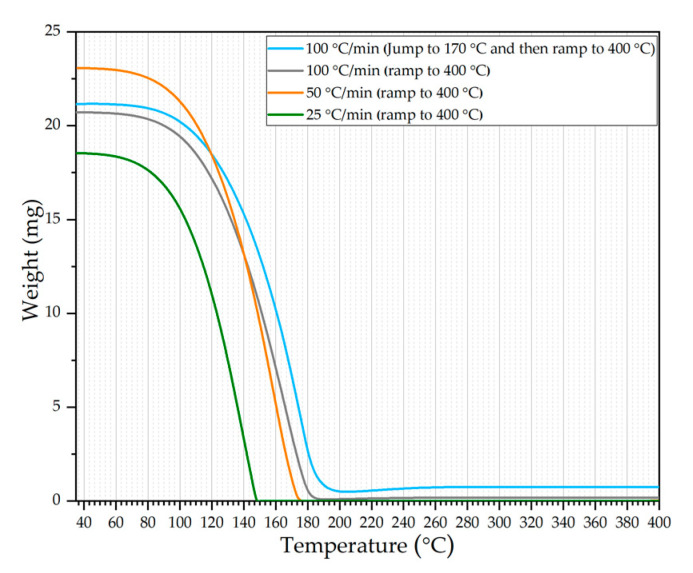
TGA curves of tungsten hexacarbonyl at different heating rates in nitrogen.

TGA was used to study the feasibility of a CVD precursor for a solution-processed method since the precursor is highly volatile and has to be converted after deposition on the substrate. In the first three experiments, the temperature was increased from 35 °C to 400 °C at different heating rates of 25 °C/min, 50 °C/min, and 100 °C/min. In the fourth experiment, the temperature was increased from room temperature to 170 °C, which is the onset of decomposition temperature of the precursor. Then, the temperature was further increased to 400 °C using a heating rate of 100 °C/min. Figure 2 shows that the precursors from four different runs started sublimating at temperatures around 65 °C and entirely sublimated when temperatures reached around 150–190 °C.

**Figure 3:**
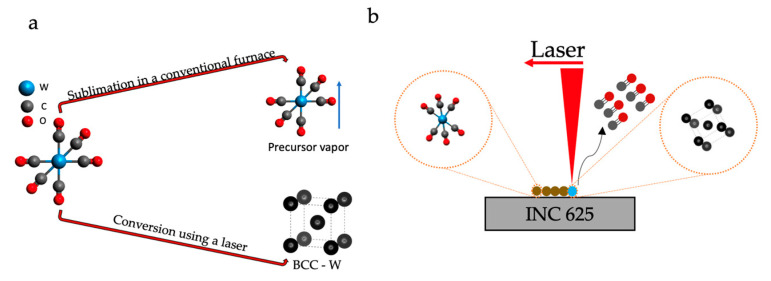
Chemistry of aerosol deposited precursor on the substrate. (**a**) transformation of precursor using different heating methods; (**b**) laser-induced decomposition of tungsten hexacarbonyl precursor.

Additionally, a blue-colored substance was observed on the TGA pan and basket of the fourth run, in which the temperature was ramped up to the decomposition temperature. This color is due to the decomposition of the precursor and the formation of WO_X_ [18]. The oxygen source might be from the regular grade nitrogen used in the experiments. Since heating was carried through a conventional furnace in TGA, information typically obtained from a TGA curve could not be seen. From the TGA curves, the precursor slowly sublimated, starting around 60 °C. Once the temperature was >100 °C, the sublimation rate was rapid. Therefore, the precursor deposited on the substrate baked in a regular furnace sublimed instead of decomposing. If the furnace temperature was >170 °C, they sublimed from the substrate and deposited on the hot walls (Figure 1b). Since the precursor vapor pressure is very high, the decomposition dominated the sublimation if the decomposition temperatures were reached very quickly. This can be achieved using a laser for heating the deposited volatile precursor because laser heating rates are very high [19]. Hence, when the laser is incident on the precursor, decomposition temperature can reach within microseconds locally, forming W before it sublimates (Figure 3a,b). 

### 3.2. Crystal Structure, Morphology, and Composition

**Figure 4:**
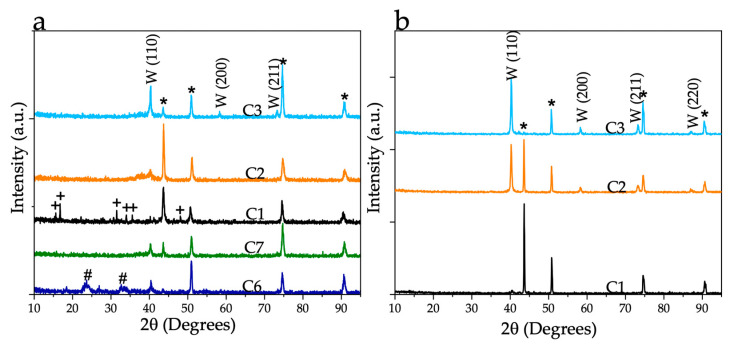
XRD of the samples converted at different energy densities: (**a**) as-converted samples and (**b**) heat-treated in vacuum at 900 °C for 1 h (* denotes Inconel 625 substrate; # denotes WO_x_ peaks; + denotes W(CO)_6_ peaks).

Figure 4a,b show the XRD patterns of the as-converted precursor using different laser beam powers, different oxygen content in the laser canister, and heat-treated samples at 900 °C for 1 h in the vacuum. As-converted samples show α-W (BCC) peaks (01-089-4900). Based on the XRD data, the presence of precursor peaks (Supplementary Figure S1) indicates that the C1 sample was not fully converted. However, the samples using the higher laser powers show complete conversion. This result suggests that the laser beam power of 60 W was not high enough for the complete conversion of the precursor. In addition, coatings consisting of W/WO_X_ were obtained when the O_2_ concentration in the canister was maintained at 21 vol%, while no WO_X_ was detected when the O_2_ content of 1.8 vol% was used. This confirms that hard vacuum (10^−6^ torr) conditions [1] are not necessary to obtain W metallic coatings, which is beneficial for the scalable production of the materials for industries. The samples C1, C2, and C3 annealed in the vacuum at 900 °C showed an increase in the intensity of W peaks. This is due to an increase in the crystallinity of the coatings. Ni-W intermetallic phases were not detected in XRD. Annealed samples were characterized to determine the morphology and composition of the W nanostructures.

(1) $$W{\left(\mathrm{CO}\right)}_{6}\;\to \;W+6\;\mathrm{CO},$$

(2) $$2\;\mathrm{CO}\;⇌{\;\mathrm{CO}}_{2}+C$$

In addition to the above reaction pathways, CO disassociation can occur and form C and O atoms at lower temperatures. The reformation of CO occurs only at higher temperatures. Another reaction pathway is the disproportionation reaction (Boudouard reaction), in which CO converts to CO_2_ and C at a temperature <700 °C. However, when the temperature is >700 °C, the backward reaction is thermodynamically favorable [20]. In previous work [3], films deposited at temperatures <400 °C contained C and O impurities due to CO disassociation at lower temperatures. When the deposition temperature was >540 °C, fewer impurities were trapped in the films. This is attributed to the less favorable nature of CO disassociation into C and O at higher temperatures. Additional heat treatment of samples at more elevated temperatures further reduced the impurities. This is due to the formation and evolution of CO from C and O impurities in the films at higher temperatures [3]. Using laser heating can reduce the impurities as laser energy produces very high temperatures within microseconds. The CO gas combustion was observed in the canister during a laser scan, which resulted in soot formation on the quartz glass. The soot formation is due to the temperature drop of CO as it leaves the substrate and is combusted into CO_2_ and elemental C. This observation implies that CO disproportionation occurred rather than disassociation after leaving the substrate.

**Figure 5:**
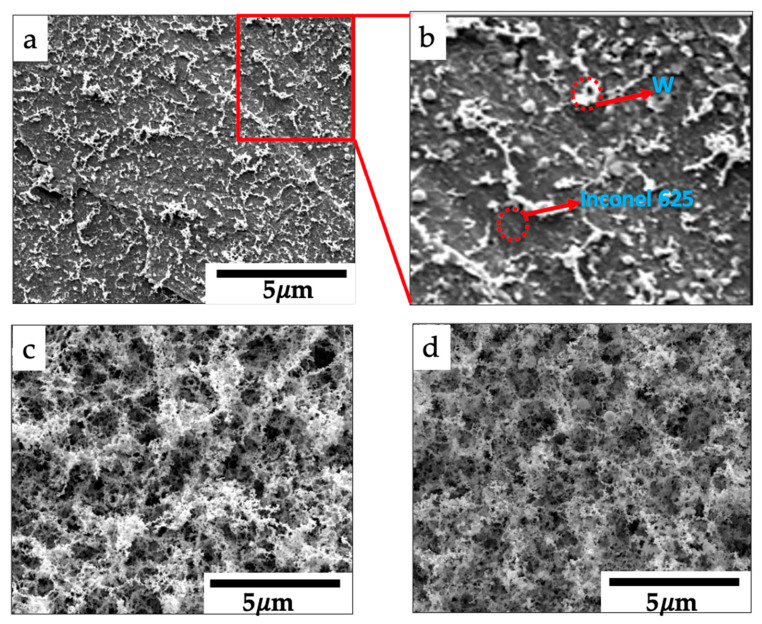
Surface morphology of samples converted at different laser beam powers and heat-treated at 900 °C using SEM: (**a**) C1, (**b**) zoomed in micrograph of C1(highlighted area), (**c**) C2, and (**d**) C3.

**Figure 6:**
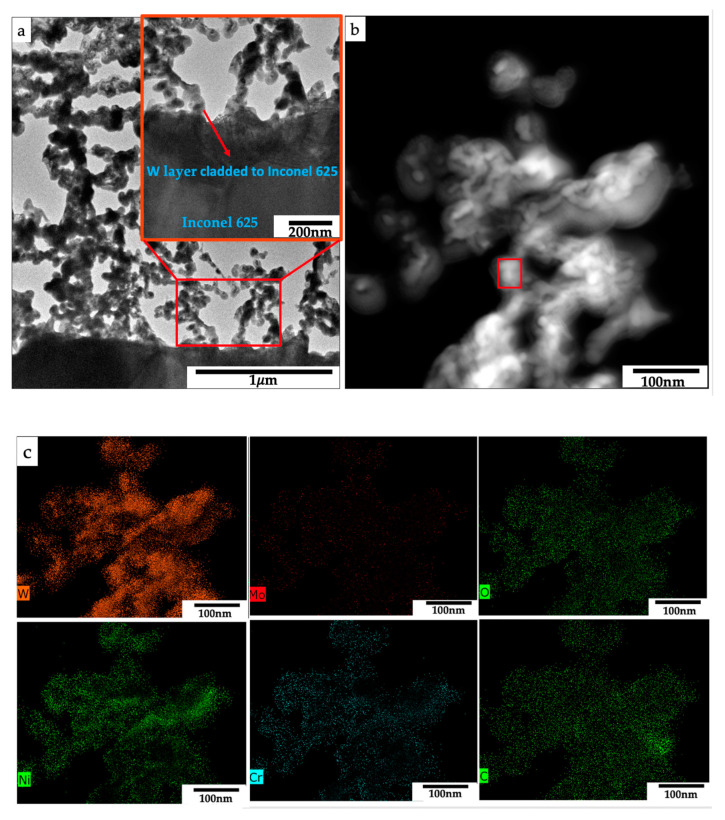
(**a**) TEM micrographs of Sample C2 showing sintered nanostructure and the cladded W nanostructures to the Inconel 625 substrate; (**b**) STEM micrograph of W network at higher magnification; (**c**) STEM-EDS elemental mapping of sample C2.

In previous studies [3], the W films produced by CVD tended to form β-W (A15) at a lower temperature. The SEM and TEM micrographs shown in Figure 5 and Figure 6 show that W nanoparticles are sintered and formed porous nanostructured layer, indicating that temperatures achieved very close to half of the melting point of W (~1700 °C). This result explains why α-W (BCC) is formed rather than the metastable β-W (A15). During the decomposition, CO can also act as a reducer. Thus, no WO_x_ peaks were observed when 1.8 vol% oxygen was used in the canister. The formation of blue WO_X_ and W in sample C6 was due to the O_2_ deficiency [18].

The SEM micrographs of the heat-treated samples converted at different laser powers are shown in Figure 5. The film showed a rough and porous network structure. Furthermore, the converted W with the laser power of 60 W had sparser W nanostructures on the substrate (Figure 5a,b) than the samples, C2 and C3, processed at higher laser powers (Figure 5c,d). This was also visually observed after the experiment. After taking it out from the laser canister, the coating on the C1 had a mixture of precursor (white color) and tungsten (black). This precursor was sublimated during the heat treatment because of its very high vapor pressure, and as a result, sparser nanostructures were seen on the micrograph (Figure 5a). This reveals that it was a partial conversion and incomplete reaction; the temperature of the substrate for sample C1 was much lower than those of the other two samples as a result of different laser parameters.

Further, TEM/STEM was used to analyze the heat-treated sample C2. The sample was prepared by FIB lift-out. C and Pt layers were deposited over the film (for protection). Figure 6a shows the cross-sectional micrograph of the converted W cladded onto the Inconel 625 substrate. Nanoparticles were generated with voids and sintered to form a continuous network of nanostructures. The converted W nanostructures were not melted but partially sintered during the laser cladding, supported by the structure with a high void fraction as shown in TEM (Figure 6a) due to the high melting temperature.

Furthermore, STEM-EDS elemental maps were obtained to investigate the distribution of the elements within the network structures. Based on Figure 6b,c and Table 2, the Inconel 625 elements were also homogeneously found from the W nanostructures. This indicates that the generated W particles likely circulated in the melt pool of Inconel 625 as a result of Marangoni flow [21]. During Marangoni flow, the melt wetted the W networks, and then the W nanostructure rose to the top surface. This is because of the lower density of the W nanoparticles, due to its porous nanostructured network, compared with the melt pool. In addition, 2.9% of W was observed in the Inconel 625 matrix. This might be due to the small amount of W particles fully melted and remained in the matrix because of their high density (Supplementary Figure S2 and Table S1). Carbon percentage may not be solely from our sample as the e-beam carbon layer and ion beam carbon layers were deposited before the Pt layer to protect the sample.

### 3.3. UV-VIS-NIR Reflectance and Solar Absorbance

**Figure 7:**
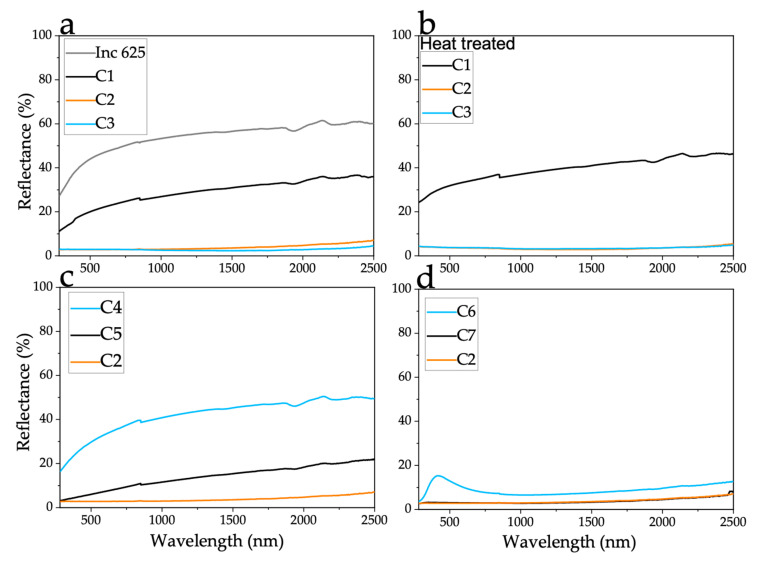
UV-VIS-NIR reflectance of samples. (**a**) as-converted samples at different laser beam power, (**b**) heat-treated samples at 900 °C for 1 h, (**c**) as-converted samples with different amounts of precursor deposited, (**d**) as-converted precursor at different O_2_ concentrations.

**Figure 8:**
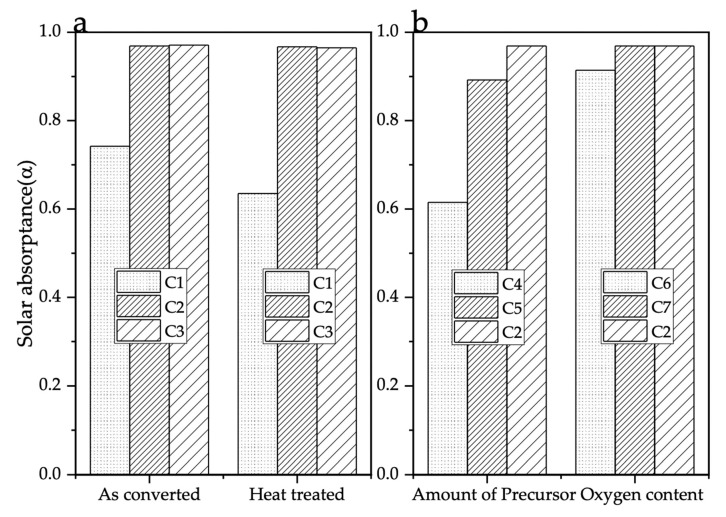
Solar absorptance of coated samples manufactured at different experimental conditions. (**a**) as-converted and heat-treated samples, (**b**) as-converted samples with different precursor amounts and at different O_2_ concentrations.

The UV-VIS-NIR reflectance of the samples was measured over the wavelength of 280–2500 nm. The solar absorptance was calculated by the weighted fraction of solar absorption of the material and incident solar radiation. The solar absorption was calculated for all the samples using the ASTM G173-03 reference spectra [22]. Figure 7 and Figure 8 show the reflectance of the samples and solar absorptance value of samples at different experimental conditions, respectively. The reflectance of samples was reduced compared to bare Inconel 625 substrate. This can be attributed to the sub-wavelength structures of W formed on the Inconel 625 substrate surface, creating a surface plasmon absorption, thereby enhancing the solar absorptance [9]. The solar absorptance of bare Inconel 625 substrate was 48.8%, and C2 and C3 were almost the same at about 96.9% and 97.2% before and after heat treatment at 900 °C in vacuum for 1 h, respectively. The solar absorptance of as-converted sample C1 was 74.2% and decreased to 63.5% after heat treatment. The coating on the as-converted C1 sample was a mixture of W and unconverted precursor. This unconverted precursor was sublimated after heat treatment resulting in coatings with less area covered by the W layer. Consequently, solar absorptance is the average of both the coating and substrate.

(3) $$\alpha =\frac{\int_{280\;\mathrm{nm}}^{2500\;\mathrm{nm}}\left(1-R\left(\lambda \right)\right)\;I\left(\lambda \right)\;d\lambda }{\int_{280\;\mathrm{nm}}^{2500\;\mathrm{nm}}I\left(\lambda \right)\;d\lambda },$$

With the different amounts of precursor ink, the thickness of W coating varies, and with 0.1 ml ink, the coating was not uniform relative to the 1 mL ink. The absorptance of sample C4 was 61.5%, and that of C5 was 89.2%. Further experiments are needed to optimize the thickness to better make the coatings cladded to the Inconel surface (only C4 has better adhesion, and the remaining can be washed away) and improve solar absorptance. The effect of O_2_ concentration was also studied on the conversion and solar absorptance. At the atmospheric O_2_ content (21 vol%), the coating contained a mixture W/WO_x_, and the solar absorptance dropped to 91.4%. However, with 1.8 vol% O_2_, the solar absorptance was almost the same as the samples converted in an inert atmosphere. This experiment shows that high solar absorptance coatings could be manufactured at ambient pressures, which is advantageous to large-scale production.

The IR emissivity of samples was not measured in this work since the solar absorptance is more important. The emissivity effect was not significant at practical receiver temperatures but could become significant at very high temperatures [23]. However, W is an inherent IR reflector, and previous studies [4] show that the emissivity of SS substrates decreased with increasing W thickness. The deposited coatings (sample C2) in this work were relatively thick (about 5–6 µm, Supplementary Figure S3), and by controlling the amount of precursor and layer by layer deposition and conversion, the adhesion and thickness can be further optimized for better solar absorptance and low IR emissivity.

## 4. Conclusions

Nanostructured W coatings were manufactured by laser conversion of tungsten hexacarbonyl on Inconel 625 substrates under an argon environment at ambient pressure. The results show that a network of black W nanostructures was obtained. STEM/EDS revealed the presence of Inconel 625 elements on W structures. XRD analysis revealed the formation of α-W at higher laser powers and W/WOx peaks with an oxygen concentration of 21 vol% and only W peaks when the oxygen concentration was 1.8 vol% and 0.5 ppm was used in the laser canister. About 97% solar absorptance was achieved with W coatings of 5–6 µm and varied with the amount of precursor deposited, uniformity of the layer, and oxygen concentration in the canister during laser conversion. Future work is needed to improve the process by uniformly depositing precursor, controlling thickness per layer, and further optimizing laser parameters to get better cladding to the substrate. Functional alloys with tailored solar absorption can be additively manufactured by integrating precursor ink chemistry with a hybrid metal additive manufacturing process. The main advantage will be to manufacture such structures in one single step without the need for post-processing. Additionally, by voxel-controlling the amount of precursor, functionally graded structures can be manufactured with specific target properties. 

## Figures and Tables

**Table 1:** Manufacturing conditions.

Sample	Laser Power (W)	Scan Speed (mm/s)	Atmosphere	Amount of Precursor Ink Deposited (mL)
C1	60	1000	Ar	1.0
C2	200	1000	Ar	1.0
C3	400	1000	Ar	1.0
C4	200	1000	Ar	0.1
C5	200	1000	Ar	0.35
C6	200	1000	21 vol% O_2_	1.0
C7	200	1000	1.8 vol% O_2_/Ar	1.0

**Table 2:** Elemental composition of the W nanostructures (highlighted area in Figure 5b).

Elements	W	Ni	Cr	Mo	Fe	Ta	Nb	Si	O	C	Mn	Al	Co	Ti	Pt
Mass%	52.2	7.33	3.08	2.18	1.76	7.95	2.28	2.96	4.30	6.66	1.16	1.45	1.45	0.63	4.55
At%	35.2	10.11	4.92	2.31	2.55	5.51	2.57	6.12	9.67	9.02	1.70	4.06	2.04	1.09	3.08

## Data Availability

Data is presented within the article.

## Supplementary Materials

The following are available online at https://www.mdpi.com/article/10.3390/nano11092304/s1, Figure S1: XRD of tungsten hexacarbonyl precursor, Figure S2: STEM/EDS of a cross-section, Table S1: Elemental composition of Inconel 625 just below the interface, Figure S3: Cross-section of the tungsten coating.
