# Supplementary material for: A Scalable Solution Route to Porous Networks of Nanostructured Black Tungsten
Source: Nanomaterials (Basel). 2021 Sep 5;11(9):2304. doi: 10.3390/nano11092304 (PMC8465037; doi:10.3390/nano11092304)
Supplement: Supplementary file 1 [file nanomaterials-11-02304-s001.zip › nanomaterials-1366455-SI.pdf]

nanomaterials

Article

A Scalable Solution Route to Porous Networks of Nanostructured Black Tungsten

V. Vinay K. Doddapaneni [1], Kijoon Lee [2,3], Tyler T. Colbert [1], Saereh Mirzababaei [2,3], Brian K. Paul [2,3], Somayeh Pasebani [2,3] and Chih-Hung Chang [1,*]

1 School of Chemical, Biological, and Environmental Engineering, Oregon State University, Corvallis, OR 97331, USA; doddapav@oregonstate.edu (V.V.K.D.); tycolbert94@gmail.com (T.T.C.)
2 School of Mechanical, Industrial, and Manufacturing Engineering, Oregon State University, Corvallis, OR 97331, USA; leekij@oregonstate.edu (K.L.); mirzabas@oregonstate.edu (S.M.); brian.paul@oregonstate.edu (B.K.P.); somayeh.pasebani@oregonstate.edu (S.P.)
3 Advanced Technology and Manufacturing Institute (ATAMI), Corvallis, OR 97330, USA
* Correspondence: chih-hung.chang@oregonstate.edu; Tel.: +1-541-737-8548

Citation: Doddapaneni, V.V.K.; Lee, K.; Colbert, T.T.; Mirzababaei, S.; Paul, B.K.; Pasebani, S.; Chang, C-H. A Scalable Solution Route to Porous Networks of Nanostructured Black Tungsten. *Nanomaterials* **2021**, *11*, x. https://doi.org/10.3390/nano11092304

Academic Editor: Ion N. Mihailescu

Received: 18 August 2021
Accepted: 31 August 2021
Published: 5 September 2021

Publisher's Note: MDPI stays neutral with regard to jurisdictional claims in published maps and institu-tional affiliations.

Copyright: © 2021 by the authors. Licensee MDPI, Basel, Switzerland. This article is an open access article distributed under the terms and conditions of the Creative Commons Attribution (CC BY) license (https://creativecommons.org/licenses/by/4.0/).

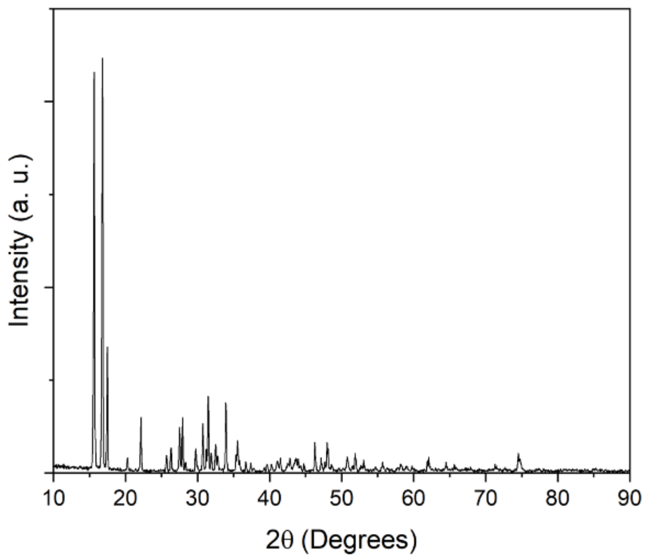

Figure S1. XRD of tungsten hexacarbonyl precursor.

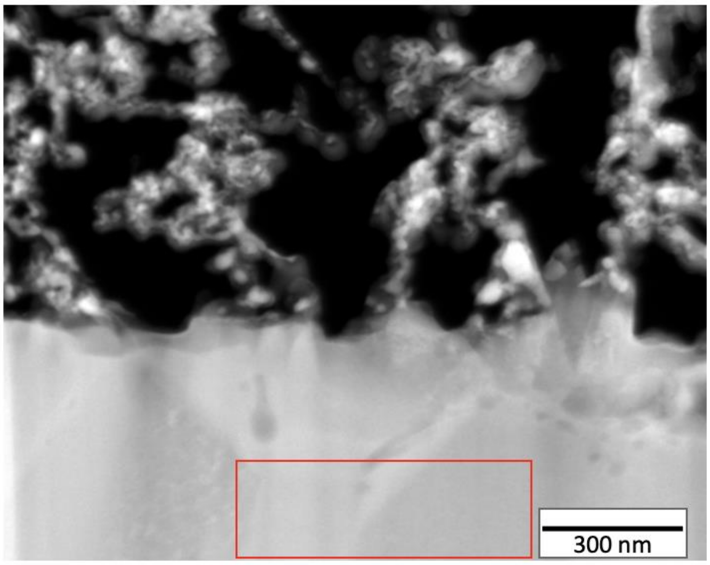

Figure S2. STEM/EDS of cross-section.

Table S1. Elemental composition of Inconel 625 just below the interface.

Elements	W	Ni	Cr	Mo	Fe	Ta	Nb	Si	O	C	Mn	Al	Co	Ti	Pt
Mass %	2.96	56.39	13.93	5.66	4.27	3.77	2.03	0.41	3.11	4.27	1.01	0.64	0.8	0.29	**0.62**

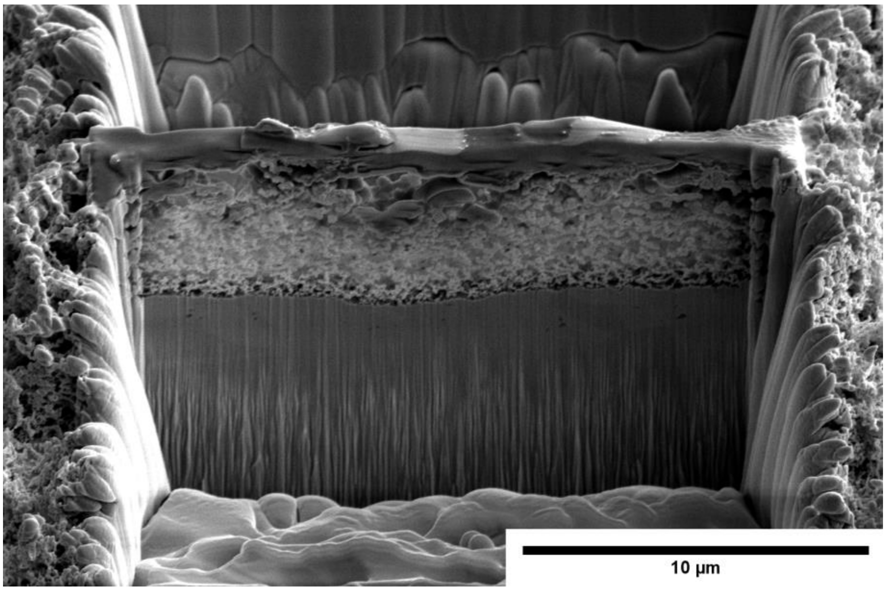

Figure S3. Cross-section of the tungsten coating.
